# Towards a Mathematical Model for the Viral Progression in the Pharynx

**DOI:** 10.3390/healthcare9121766

**Published:** 2021-12-20

**Authors:** Raj Kumar Arya, George D. Verros, Devyani Thapliyal

**Affiliations:** 1 Department of Chemical Engineering, Dr. B. R. Ambedkar National Institute of Technology, Jalandhar 144011, India; devyanithapliyal5@gmail.com; 2 Laboratory of Polymer and Colour Chemistry and Technology, Department of Chemistry, Aristotle University of Thessaloniki (AUTH), P.O. Box 454, Plagiari, Epanomi, 57500 Thessaloniki, Greece; gdverros@yahoo.gr

**Keywords:** mathematical modeling, simulation, diffusion, pharynx, virus

## Abstract

In this work, a comprehensive model for the viral progression in the pharynx has been developed. This one-dimension model considers both Fickian diffusion and convective flow coupled with chemical reactions, such as virus population growth, infected and uninfected cell accumulation as well as virus clearance. The effect of a sterilizing agent such as an alcoholic solution on the viral progression in the pharynx was taken into account and a parametric analysis for the effect of kinetic rate parameters on virus propagation was made. Moreover, different conditions caused by further medical treatment, such as a decrease in virus yield per infected cell, were examined. It is shown that the infection fails to establish by decreasing the virus yield per infected cell. It is believed that this work could be used to further investigate the medical treatment of viral progression in the pharynx.

## 1. Introduction

Viruses are inherently related to the existence of many diseases such as hepatitis C (HCV), hepatitis B (HBV), AIDS (HIV), influenza, etc. How a viral infection will progress in the human body depends on many factors, such as the rate of spread of the agent, the immune response, what treatment may be applied, etc. [1]. In order to investigate all these factors, one has to resort to mathematical models. Mathematical models, on the other hand, have received comparatively limited study. More specifically, modeling of viral infection has become an important tool contributing to a better understanding of the speed with which human immunodeficiency virus (HIV) replicates [2,3], the dynamics of different drug classes for HIV [4], the main mode of action of interferon in hepatitis C virus (HCV) [5], the effects of direct-acting antivirals in HCV [6], potential effects of the immune response [7], and many others [8,9,10,11,12,13].

The biological principles regulating dynamic variations in viral load and clinical symptoms can be understood and quantified using mathematical modeling. Parameters that measure the interactions between the virus, its host, course of a disease, and its treatment outcomes can be determined by fitting models to viral load data [14,15]. Over the last few decades, mathematical models have been used extensively in a variety of biomedical domains. Stochastic models of genetic mutations have proved useful in cancer research [16]. Additionally, in HIV/AIDS research, combining mathematical models with patient-level data yielded crucial insights into early infection dynamics, and model-based inference of key physiological parameters sped up the development of therapy regimens [17,18].

To examine the localization and transmission of influenza A virus infections in the human respiratory tract, partial differential equations were used to construct a mathematical model [19]. The primary factors relevant for the optimization of virus antigen yields, according to a mathematical model that depicts the replication of influenza A virus in animal cells in large-scale micro-carrier culture, are the specific virus replication rate and particular cell death rate owing to infection [20]. Various researchers have looked into the dynamics of influenza transmission, and some have proposed mathematical models for influenza transmission dynamics [21,22,23,24,25].

A considerable number of viruses, such as influenza, enters the human body through the respiratory system. The pharynx is the part of the throat behind the mouth and nasal cavity, and above the esophagus and larynx [26]. The pharynx can be infected by a variety of viruses. Adenovirus, Epstein-Barr virus, rhinovirus, coronavirus, influenza virus, herpes simplex virus, and para-influenza virus are the most common viruses. Viruses, after entering the nasal cavity or the mouth, progress in to the pharynx and then leach into the stomach or the lungs. It is also assumed that there is a critical virus concentration for the leaching of the virus through the larynx into the lungs.

Many viruses can replicate in the cells that line the respiratory system. Mucus and cilia on the epithelial cells protect the respiratory tract’s surface. Inhaled viruses are retained in mucus, transferred to the pharynx by ciliary motion from the airways and nasal cavity, and then swallowed or coughed up. Droplets frequently travel to the trachea and bronchioles, where they become stuck in the mucus blanket. Droplets can be inhaled directly into the lungs, and some can make it to the alveoli, where virus particles can directly infect alveolar epithelial cells, resulting in viral pneumonia [27].

The aim of this work is to investigate how a virus progresses in the human pharynx by using simple comprehensive mathematical modeling. This work is organized as follows: in the theoretical section the governing equations for the spread of a virus in human pharynx along with the initial and boundary conditions are given, and in the results and discussion section, simulations are presented. Finally, conclusions are drawn and ideas on how this work could be applied to the recent pandemic of COVID-19 are presented.

## 2. Theoretical Section

The virus, after entering the first part of the pharynx, will move by both convective flows and diffusion to the lower part of the pharynx and simultaneously increase its population by infecting healthy cells. This problem from an engineering point of view is a diffusion problem coupled with chemical reactions. In particular, it was assumed that only the virus species diffuse in the pharynx. This work closely follows the extracellular model of Rawlings and coworkers [28,29]. Their extracellular model includes not only infection and virus propagation but also the accumulation of uninfected cells [28].

Moreover, convective flow of virus was considered and a virus clearance reaction was also considered, by following Quirouette et al. [19]. In particular, the following elementary chemical reactions were considered:

***Infection:***

$\mathrm{virus}\;\;+\;\;\mathrm{infected}\;\;\mathrm{cell}\;\;\overset{k_{1}}{\to }\mathrm{infected}\;\;\mathrm{cell}$

***Virus propagation:***

$\;\;\mathrm{infected}\;\;\mathrm{cell}\;\;\overset{k_{2}}{\to }Y\;\mathrm{virus}\;\;+\;\;\mathrm{dead}\;\;\mathrm{cell}$

***Virus clearance:***

$\;\mathrm{virus}\;\;\overset{k_{3}}{\to }\;\mathrm{deactivated}\;\mathrm{virus}$

***Uninfected cells accumulation:***

$\;\mathrm{Substrate}\;+\;\mathrm{uninfected}\;\mathrm{cell}\;\;\overset{k_{4}}{\to }\;2\;\mathrm{uninfected}\;\;\mathrm{cells}$

Based on the above mechanism, the resulting mass balances are written as follows [19,28,29]:

(1) $$\frac{\partial C_{vir}}{\partial t}=\frac{\partial }{\partial z}\left(D\frac{\partial C_{vir}}{\partial z}\right)-k_{1}C_{vir}C_{unc}+Yk_{2}C_{\inf c}+v_{0}\frac{\partial C_{vir}}{\partial z}-k_{3}C_{vir}$$

(2) $$\frac{\partial C_{\inf c}}{\partial t}=k_{1}C_{vir}C_{unc}-k_{2}C_{\inf c}$$

(3) $$\frac{\partial C_{unc}}{\partial t}=-k_{1}C_{vir}C_{unc}+2k_{4}C_{sub}C_{unc}$$

where *D* stands for virus diffusion coefficient, *C* represents various species concentration, *t* is the infection time, *v*_0_ is the convective flow velocity, and *z* stands for axial distance. Subscript *infc* represents infected cells and subscript *unc* represents uninfected cells. Subscripts *vir* and *sub* represent the virus and the substrate, respectively.

The positive sign of the virus convective term shows that the virus physically moves upwards with velocity *v*_0_. By introducing the fraction of virus concentration with respect to the initial virus concentration *u*_1_ (*u*_1_ = *C_vir_/C_unc0_*), the dimensionless position *η* (*η* = *z/L*_0_), the dimensionless time *τ* (*τ = D*${}_{0}$*t/L*_0_^2^), the ratio of infected cells to initial cells *X*_1_ (*Χ*_1_ *= C_infc_/C_unc0_*), and the ratio of uninflected cells *X*_2_ (*Χ*_2_ *= (C_unc_/C_unc_*_0_), the above equations are written as:

(4) $$\frac{\partial u_{1}}{\partial \tau }=\frac{\partial }{\partial \eta }\left(\frac{D}{D_{0}}\frac{\partial u_{1}}{\partial \eta }\right)+\frac{L_{0}^{2}}{D_{0}}\left(-k_{1}c_{unc0}u_{1}X_{2}+Yk_{2}X_{1}-k_{3}u_{1}\right)+\frac{L_{0}^{}}{D_{0}}v_{0}\frac{\partial u_{1}}{\partial \eta }$$

(5) $$\frac{\partial X_{1}}{\partial \tau }=\frac{L_{0}^{2}}{D_{0}}\left(k_{1}c_{unc0}u_{1}X_{2}-k_{2}X_{1}\right)$$

(6) $$\frac{\partial X_{2}}{\partial \tau }=\frac{L_{0}^{2}}{D_{0}}\left(-k_{1}c_{unc0}u_{1}X_{2}+2k_{4}C_{sub}X_{2}\right)$$

where *L*_0_ stands for pharynx length, *D*_0_ is a scaling parameter with the diffusion coefficient as a unit, and subscript 0 represents initial conditions. One could directly derive Equations (4)–(6) from Equations (1)–(3) by using the dimensionless quantities previously introduced.

In this work, the pharynx extends from the nasal cavity (*η* = 0) up to the larynx (*η* = 1). The following initial and boundary conditions were considered:

(7) $$D\frac{\partial u_{1}}{\partial \eta }=0\;\;\;\;\;\;\;\;\;\;\;\eta =0;\;\eta =1$$

*u*_1_ = *u*_10_                *τ* = 0 (8)

*Χ*_1_ = 0, *X*_2_ = 1         *τ* = 0 (9)

The most adverse case of a closed system after infection was considered by applying Equation (7). Alternatively, open boundary conditions [30] could be applied at the end of the computational domain.

The governing Equations (4)–(6) along with the initial and boundary conditions (7)–(9) were simultaneously solved by using the Galerkin Finite Element Method (GFEM). The system of algebraic equations resulting after weighting with quadratic basis functions and applying the divergence theorem as well as the boundary conditions was numerically solved by Newton’s method. A homemade code written in FORTRAN was utilized for the simulations. A complete description of the method is given in full detail elsewhere [31,32,33]. Although more sophisticated models, including population balances, which take into account the effect of the age of infected cells on virus production, are available in the literature, Rawlings and co-workers [28,29] have shown that simple extracellular models as applied in this work are still applicable.

## 3. Results and Discussion

In this work, the pharynx length (*L*_0_) was set equal to 0.15 m, which is a typical value for an adult. The scaling parameter *D*_0_ was set equal to 10^−7^ m^2^/s. To account for convective flows in the pharynx, the velocity *v*_0_ was set equal to 40 μm/s [19]. The virus diffusion coefficient was assumed equal to 10^−12^ m^2^/s, which is in close agreement with the values for this coefficient reported by other workers in the field [19,28,29]. The value for *u*_10_ was set equal to 7.5 × 10^−2^ for the whole computational domain [19]. Following Rawlings and co-workers [28,29], the kinetic rate parameters, *k*_1_*C_unc_*_0_ and *k*_2_ were set equal to 1.4 × 10^−6^ s^−1^ and 1.6 × 10^−5^ s^−1^, respectively. The *C_unc_*_0_ was set equal to 3.8 × 10^7^ cells/cm^3^ [28].

The computational domain was discretized in 50 elements equally distributed along the pharynx. The length of each element was 0.3 cm. The time step was set equal 10^−6^. Any additional mesh refinement or step decrease has no practical effect on the model predictions.

The aim of this work is to study the progression of the virus in the pharynx by using a minimum number of adjustable parameters. Therefore, it was assumed for simplicity that the concentration of the substrate (*C_sub_*) is a constant. This allows us to use a combined kinetic rate constant (*k*_4_*C_sub_*) for uninfected cells’ accumulation reaction.

The following values for the Y, *k*_3_, and *k*_4_*C_sub_* were adopted by assuming an aggressive virus such as influenza A virus. In particular, these parameters were adjusted in such a way that a value of viral relative load (*u*_1/_*u*_10_) of eight orders of magnitude is reached within three days after infection. Moreover, a reduction of relative virus load (*u*_1/_*u*_10_) to six orders of magnitude at six days after infection was considered. Finally, a value of viral relative load (*u*_1/_*u*_10_) of three orders of magnitude at one day after infection was also considered. The resulting values for the Y, *k*_3_, and *k*_4_*C_sub_* are 21.4, 1.25 × 10^−4^ s^−1^, and 10^−4^ s^−1^, respectively. The value for *k*_3_ is in the same order of magnitude with the reported value by Quirouette et al. [19] (6.1 × 10^−5^ s^−1^). The reported value in this work for Y: 21.4 could be surprising at first glance; this relatively high value could be attributed to the fact that the same population of cells is present during the whole infection time, or in other words, death of cells is caused not only by infection but also by the inherent differences in the parameters of various literature models.

**Figure 1:**
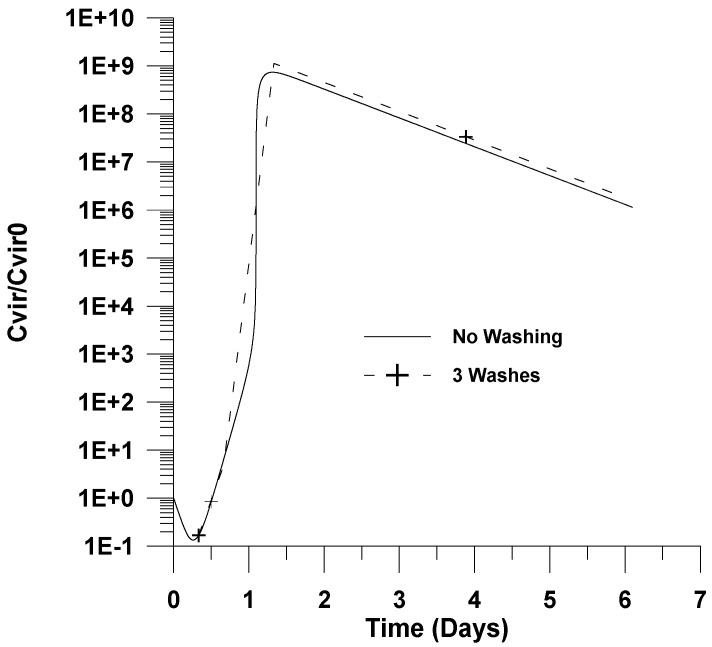
Effect of washing on dimensionless virus concentration (*C_vir_*/*C_vir_*_0_ = *u*_1/_*u*_10_). Virus removal of 99% per washing, three washings, *u*_10_ = 7.5 × 10^−2^.

**Figure 2:**
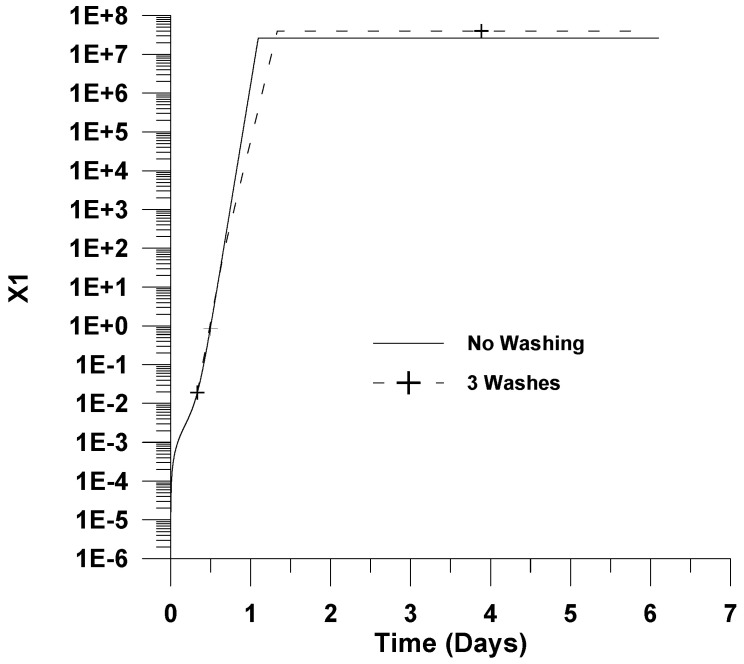
Effect of washing on infected cells’ dimensionless concentration (*X*_1_). Virus removal of 99% per washing, three washings, *u*_10_ = 7.5 × 10^−2^.

**Figure 3:**
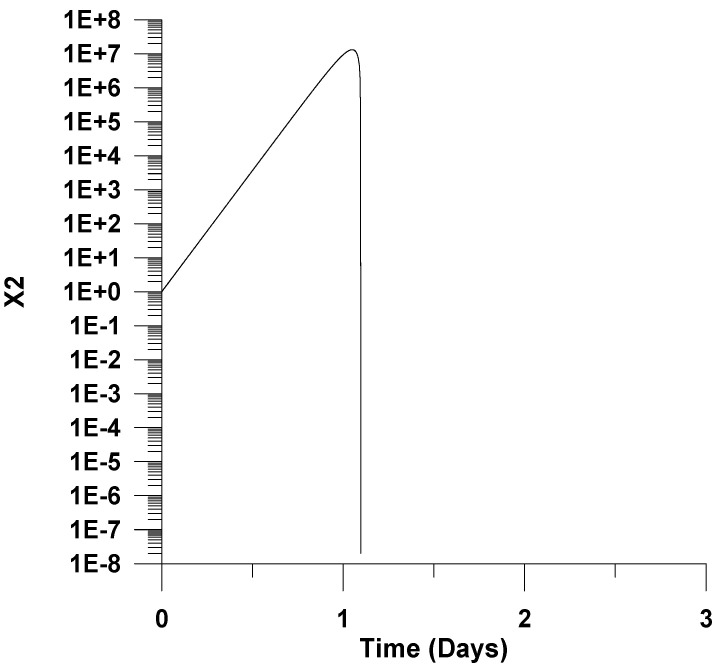
Uninfected cells’ dimensionless concentration (*X*_2_) as a function of time, *u*_10_ = 7.5 × 10^−2^.

The simulations obtained are depicted in Figure 1, Figure 2 and Figure 3. The local maxima with respect to position obtained at each time are presented throughout this work as a function of infection time.

In Figure 1 and Figure 2 the effect of washing with a sterilizing agent on viral progression is also illustrated. In these figures, for presentation reasons only, the local maxima of the dimensionless virus concentration with respect to time are plotted for the sterilizing agent case. Typical antiseptics for the pharynx include alcoholic solutions or other small molecules solutions, such as sodium fluoride (NaF). It is assumed that 99% of the virus is deactivated after each washing. This value is typical for treatment with sterilizing solutions such as alcohol 70% (*w*/*v*) for short washing times [34]. Lower concentrations of alcohol in the solution could also be considered by increasing the washing time.

Figure 1 depicts the effect of washes with a sterilizing agent on the dimensionless virus concentration. It is shown in Figure 1 and Figure 2 that washing the pharynx with a sterilizing agent has no effect on the infection. This could be attributed to the fact that the virus concentration after clearance by a sterilizing agent rapidly increases due to the relatively high rate of infection. The results presented in Figure 1 for the infection without using a sterilizing agent are in accordance with the results found in the literature [19,21].

Figure 2 and Figure 3 depict *X*_1_ and *X*_2_ as a function of infection time. It is shown that *X*_1_ and *X*_2_ initial exponentially increase with infection time until the point of maximum virus concentration (please, see Figure 1). The *X*_1_ remains almost constant after this point, while *X*_2_ sharply decreases, indicating that all cells are infected.

A high accumulation of uninfected cells up to seven orders of magnitude is observed in Figure 3; this discrepancy could be attributed to the inclusion of uninfected cells accumulation reaction. Attempts made by the authors to avoid uninfected cells’ accumulation reaction by only considering the rest reactions or even by considering a constant concentration of uninfected cells result in an extremely high value of Y (virus yield per infected cell) or in a failure to capture the fundamental trends of the literature for virus infection in the pharynx [19,21]. This could be viewed as a limitation of the applied model. Please note that our task is to study the infection in the pharynx by keeping the number of adjustable parameters to a minimum value. Therefore, additional reactions were not further considered. However, simple extracellular models as applied in this work; moreover, their limitations could still be used to elucidate virus dynamics and cells infection [28].

**Figure 4:**
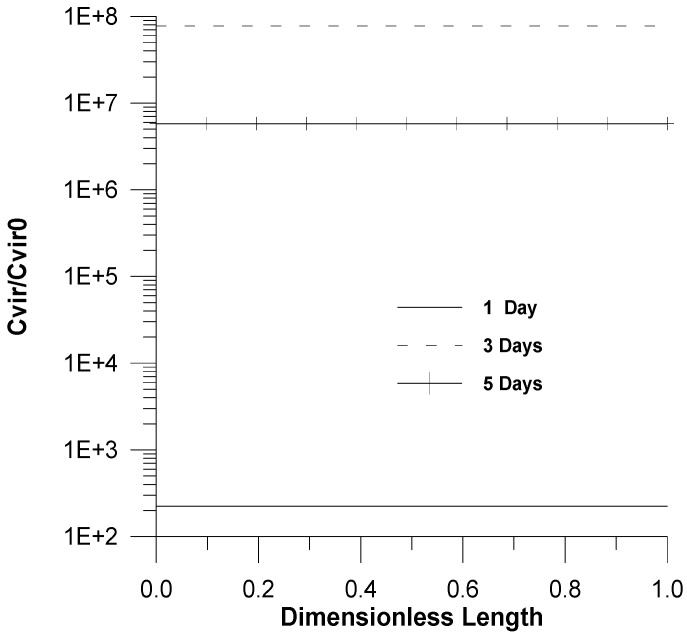
Profiles of the dimensionless virus concentration (*C_vir_*/*C_vir_*_0_ = *u*_1/_*u*_10_) as a function of time, *u*_10_ = 7.5 × 10^−2^.

**Figure 5:**
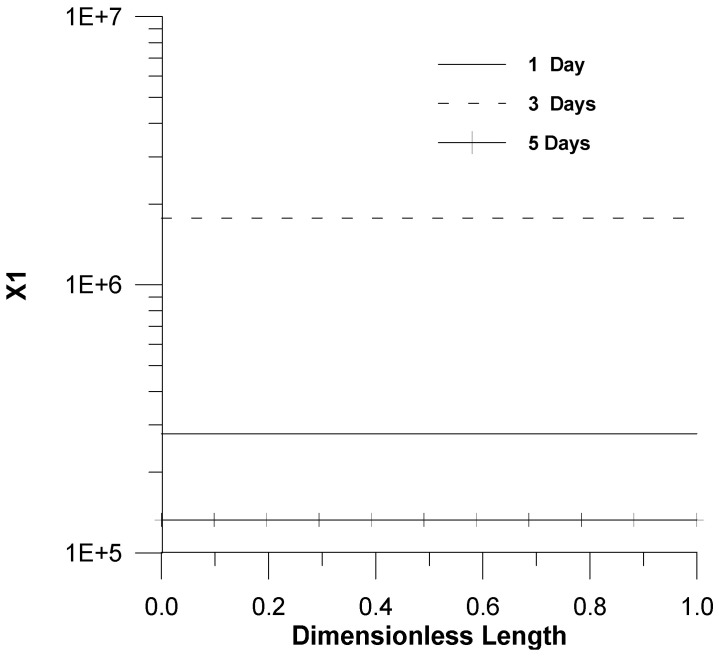
Profiles of infected cells’ dimensionless concentration (*X*_1_) as a function of time, u_10_ = 7.5 × 10^−2^.

In Figure 4 and Figure 5, smooth linear profiles in the pharynx for the dimensionless virus concentration and X_1_ are shown. This is attributed to the fact not only that a uniform initial infection by a virus was considered but also to the fast dynamics of chemical reactions.

**Figure 6:**
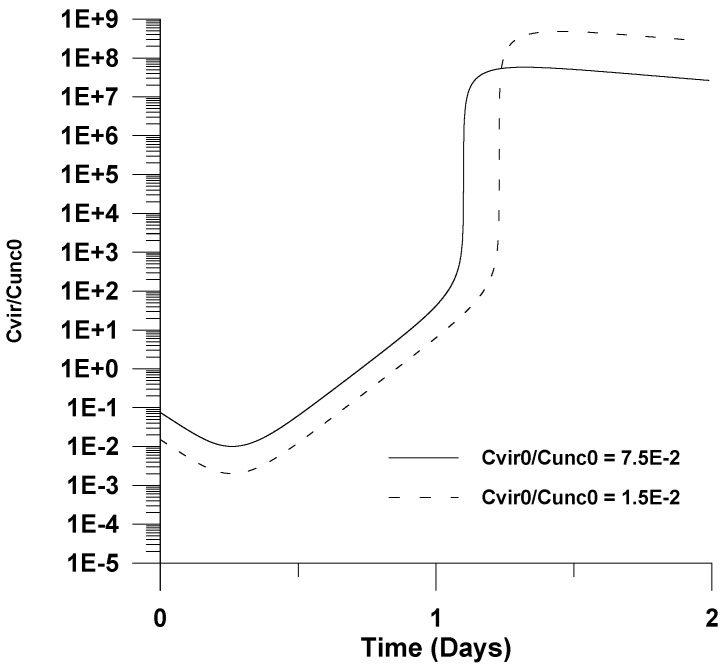
Effect of the initial virus concentration (*u*_10_) on virus concentration (*u*_1_ = *C_vir_*/*C_unc_*_0_).

**Figure 7:**
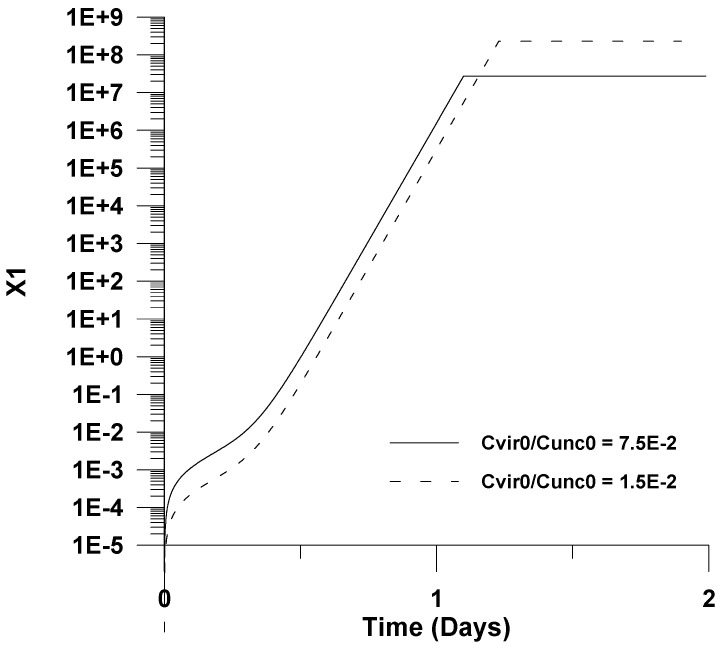
Effect of the initial virus concentration (*u*_10_) on infected cells’ dimensionless concentration (*X*_1_).

The effect of the initial virus concentration on the virus concentration (*u*_1_ = *C_vir_*/*C_unc_*_0_) and *X*_1_ is illustrated in Figure 6 and Figure 7. It is shown that as the initial virus concentration decreases, the maximum virus concentration with respect to infection time increases; this is surprising from first glance. However, this effect could be attributed to the fact that a lower initial virus concentration leads to a delay of virus clearance. This effect coupled with the uninfected cells accumulation reaction effects could cause not only a delay of maximum infection with respect to infection time, but could also lead to higher virus concentrations.

**Figure 8:**
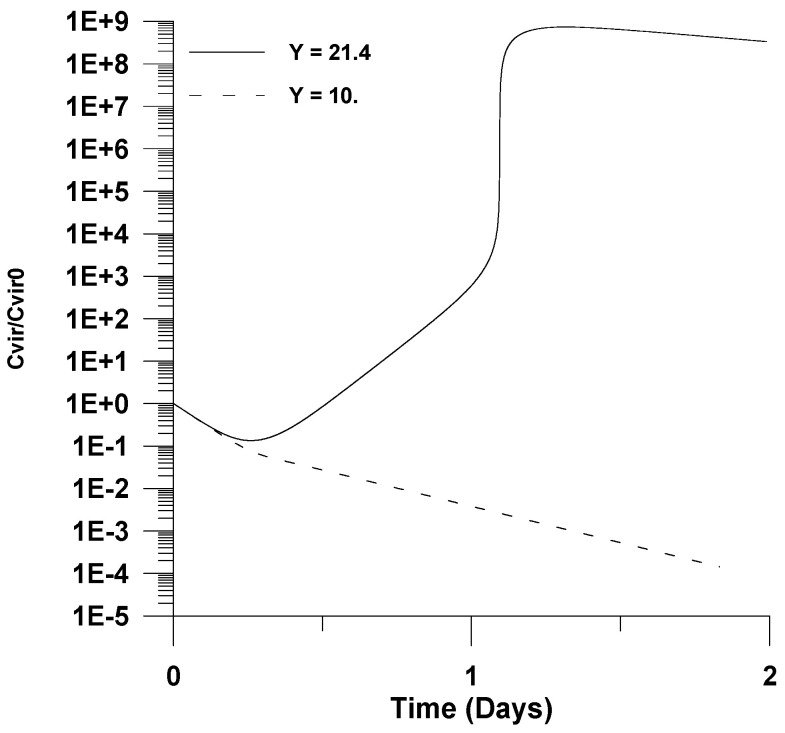
Effect of the virus yield per infected cell (Y) on the dimensionless virus concentration (*C_vir_*/*C_vir_*_0_ = *u*_1/_*u*_10_), *u*_10_ = 7.5 × 10^−2^.

The main effect of decreasing the yield of virus per infected cell is depicted in Figure 8. More specifically, it is shown that the infection fails to establish by decreasing the virus yield by almost half.

**Figure 9:**
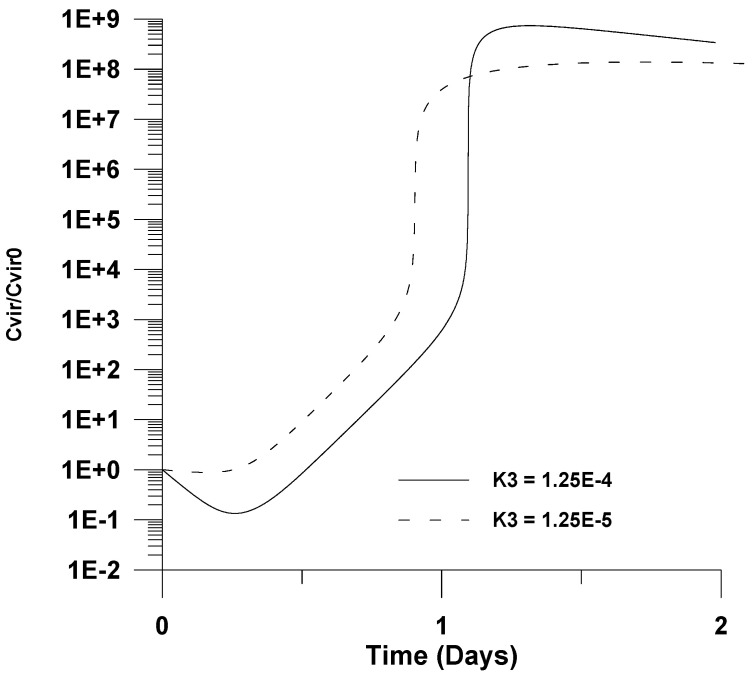
Effect of the virus clearance kinetic rate constant (*k*_3_) on the dimensionless virus concentration (*C_vir_*/*C_vir_*_0_ = *u*_1/_*u*_10_), *u*_10_ = 7.5 × 10^−2^.

**Figure 10:**
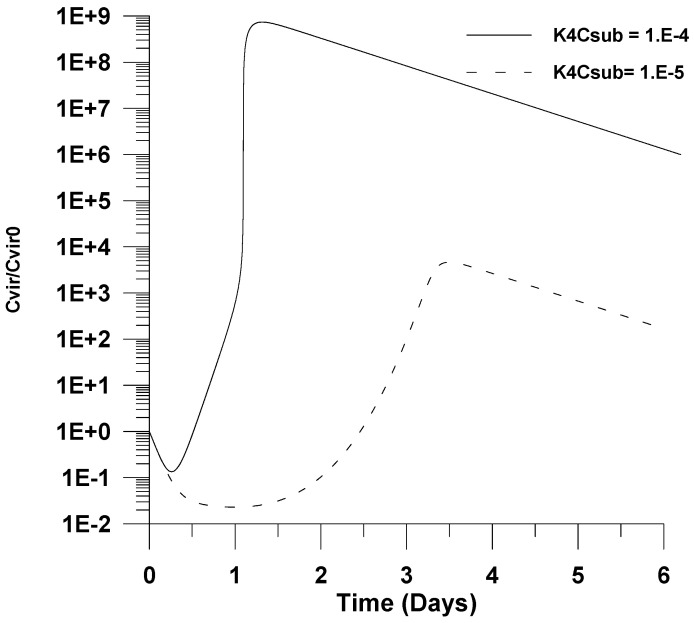
Effect of the uninfected cells’ accumulation kinetic rate constant (k_4_) on the dimensionless virus concentration (*C_vir_*/*C_vir_*_0_ = *u*_1/_*u*_10_), *u*_10_ = 7.5 × 10^−2^.

**Figure 11:**
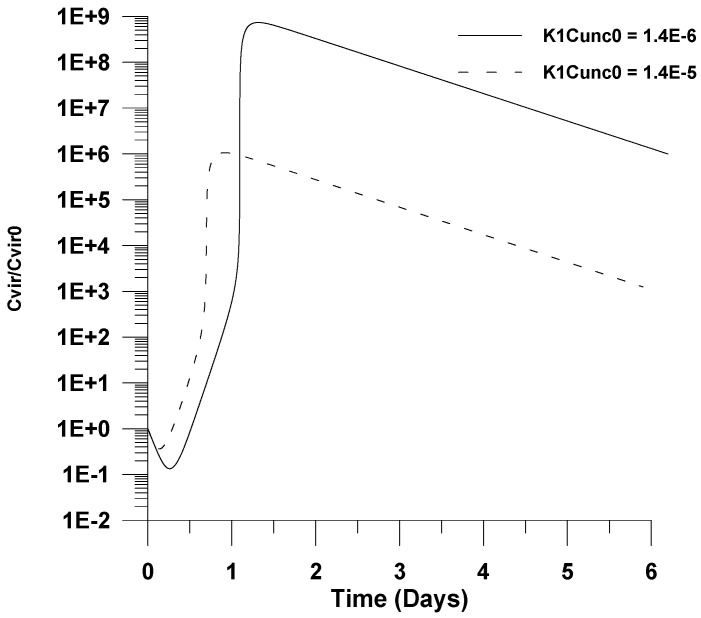
Effect of the virus infection combined kinetic rate constant (*k*_1_*C_m_*_0_) on the dimensionless virus concentration (*C_vir_*/*C_vir_*_0_ = *u*_1/_*u*_10_), *u*_10_ = 7.5 × 10^−2^.

**Figure 12:**
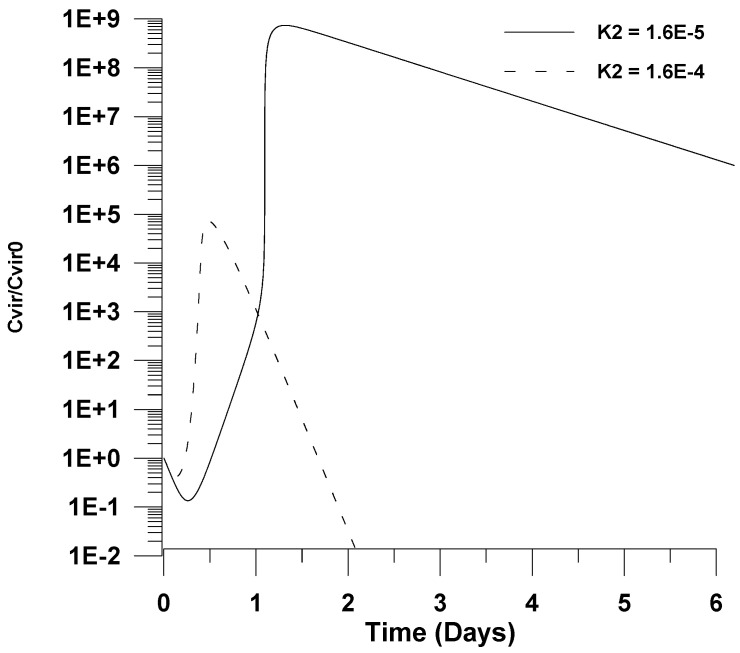
Effect of the virus propagation kinetic rate constant (*k*_2_) on the dimensionless virus concentration (*C_vir_*/*C_vir_*_0_ = *u*_1/_*u*_10_), *u*_10_ = 7.5 × 10^−2^.

In Figure 9, Figure 10, Figure 11 and Figure 12, the effect of various kinetic rate constants on the dimensionless virus concentration profiles with respect to infection tine is shown. In particular, Figure 9 shows that a decrease of the virus clearance kinetic rate constant (*k*_3_) by an order of magnitude has very little effect on the maximum infection. This is attributed to the fact that the maximum virus value is mainly controlled by other reactions, such as infection, virus propagation, and uninfected cell accumulation. The value of the virus clearance kinetic constant is the controlling step only in the initially steps of infection, where there is a relatively small number of infected cells (see Figure 2) and after the peak value where the number of uninfected cells is relatively small (see Figure 3).

In Figure 10, the effect of decreasing the combined kinetic rate constant for uninfected cells accumulation (*k*_4_*C_sub_*) is illustrated. It is shown that a decrease in the combined kinetic rate constant for uninfected cells accumulation (*k*_4_*C_sub_*) by an order of magnitude has a strong effect on infection. This could be explained from the fact that lower values for this kinetic rate constant could lead to smaller values of produced uninfected cells, causing a further decrease in the peak virus concentration.

Finally, in Figure 11 and Figure 12, the effect of increasing the kinetic rate constants for infection (*k*_1_*C_unc_*_0_) and virus propagation (*k*_2_) is illustrated. In particular, it is shown in both figures that an increase by one order of magnitude of each kinetic rate constant could cause a further decrease on the maximum value of virus concentration. This could be explained by the fact that the infection and the virus propagation reactions are further accelerated, causing the maximum value of the virus concentration to appear at shorter infection times, where the number of uninfected cells is rather small due to the cell accumulation reaction.

Regarding viruses having a long incubation period (days) before symptoms start to show [35], this study shows that additional medical treatment by reducing the virus yield per infected cells (Y) could be applied only in special cases, such as the recent pandemic of COVID-19.

## 4. Conclusions

In this work, a comprehensive framework for modeling viral progression in the pharynx was developed. The effect of using a sterilizing agent such as an alcoholic solution for washing the pharynx was studied using a numerical experiment. It was shown that the use of a sterilizing agent has no effect on the progress of a virus in the pharynx. Moreover, it is shown that additional treatment with a medicine causing a reduction in virus yield per infected cell has a strong effect on the virus propagation. This study could support that additional medical treatment could be applied in addition to other measures, such as protective equipment (masks, gloves, etc.), social distancing, lockdowns, etc., in order to reduce the rate of population infection, and thus, lightening the burden on health care systems in developing countries in case of a pandemic such as the COVID-19 pandemic. It is also believed that this work could be used to further investigate the medical treatment of viral progression in the pharynx.

## Data Availability

Not applicable.

## Nomenclature

C	Species concentration, cells.m^−3^
*D*	Binary diffusion coefficient, m^2^.s^−1^
*D*_0_	Scaling Factor, m^2^.s^−1^
*k*_1_	Kinetic rate constant for infection, cells^−1^.m^3^s^−1^
*k*_2_	Kinetic rate constant for virus propagation, s^−1^
*k*_3_	Kinetic rate constant for virus clearance, s^−1^
*k*_4_	Kinetic rate constant for cells accumulation, cells^−1^.m^3^s^−1^
*L*_0_	Pharynx length, m
*t*	Time, s
*u*_1_	Virus concentration, dimensionless
*u_i_*_0_	Initial virus concentration, dimensionless
*v*_0_	Convective flow velocity, m.s^−1^
*X*_1_	Ratio of infected cells to initial cells
*X*_2_	Degree of conversion of uninfected cells to infected cells
*Y*	Yield of virus per infected cell
*z*	Axial coordinate, m
**Greek Letters**	
*η*	Dimensionless length
*τ*	Dimensionless time
**Subscript**	
0	Initial Conditions
*infc*	Infected cells
*sub* Substrate	
*unc*	Uninfected cells
*vir*	Virus
